# Carpal Tunnel Syndrome as an Atypical Presentation of Chikungunya: A Case Report

**DOI:** 10.7759/cureus.65085

**Published:** 2024-07-22

**Authors:** Gurjot Singh, Shubam Trehan, Kanishka Goswami, Meet Popatbhai Kachhadia, Piyush Puri

**Affiliations:** 1 Internal Medicine, Maharaj Sawan Singh Charitable Hospital, Beas, IND

**Keywords:** viral complications, immune-mediated injury, neurological complications, carpal tunnel syndrome, chikungunya virus

## Abstract

Chikungunya virus (CHIKV) is an arbovirus endemic to tropical and subtropical regions, primarily known for causing fever and severe joint pain. However, its capacity to induce neurological complications is less frequently documented. This case report highlights a rare presentation of carpal tunnel syndrome (CTS) following CHIKV infection, expanding the spectrum of CHIKV manifestations beyond its common arthropathic effects.

We detail the case of a 45-year-old male who developed acute CTS symptoms, including pain, numbness, and motor dysfunction in the right thumb, six weeks after experiencing typical CHIKV symptoms of high-grade fever and arthralgia. Despite an initial treatment regimen of corticosteroids aimed at reducing inflammation, the patient's symptoms showed minimal improvement, prompting surgical intervention. Following carpal tunnel release surgery, the patient experienced significant relief and functional recovery. This case underscores the importance of considering CHIKV in the differential diagnosis of CTS in endemic areas, particularly when preceded by typical viral infection symptoms. It also supports surgical intervention as a viable treatment option for CTS associated with CHIKV when conservative management is ineffective, highlighting the need for an interdisciplinary approach in treating atypical manifestations of CHIKV infections.

## Introduction

Chikungunya virus (CHIKV) is an arthropod-borne virus predominantly found in South Asia and is known for causing significant and recurrent outbreaks that pose major public health challenges. The disease chikungunya, triggered by this virus, has recently garnered considerable attention as a critical health issue, particularly following major outbreaks in the Indian Ocean Islands and India. Notably, in 2006, an estimated 1.38 million individuals in southern and central India were reported to have developed symptoms of the disease, although the actual incidence may have been much higher due to potential underreporting and inaccuracies in the health reporting systems [1].

First isolated in Tanzania in 1953, CHIKV is a member of the Togaviridae family, which comprises single-stranded RNA alphaviruses and is characterized by three distinct genotypes: East African, West African, and Asian. Historical outbreaks in India, specifically those in 1963 and 1973, were caused by the Asian genotypes. However, more recent outbreaks, such as the 2005 epidemic in the Indian Ocean islands and the subsequent 2006 epidemic in India, have been primarily driven by the East African genotype [1-4].

Transmission of CHIKV to humans occurs through the bites of infected mosquitoes, specifically Aedes aegypti and Aedes albopictus. The clinical manifestations of chikungunya are generally severe, with patients typically experiencing high-grade fever and intense joint pain. Beyond these common symptoms, CHIKV can also lead to severe neurological complications. These include encephalomyelopathy, myeloneuropathy, Guillain‐Barré syndrome, acute disseminated encephalomyelitis, neonatal hypotonia, and various neuro-ocular diseases such as uveitis, retinitis, and optic neuritis [5].

Among these neurological impacts, carpal tunnel syndrome (CTS) stands out as a particularly rare and intriguing complication of CHIKV infection. The pathogenesis of CTS in the context of CHIKV is thought to be immune-mediated, likely triggered by the body’s inflammatory response to the viral infection [6]. This case report delves into a unique occurrence of CTS following a CHIKV infection, underscoring the diagnostic complexities and the challenges faced in its treatment, especially when conventional medical therapies prove insufficient. This exploration highlights the multifaceted nature of CHIKV’s clinical impact and the critical need for a deeper understanding of its broader health implications.

## Case presentation

In this case report, we describe a 45-year-old male who presented to emergency services with an acute onset of pain, numbness, tingling, and motor impairment, including weakness and clumsiness in the right thumb. The patient reported that these symptoms had emerged six weeks following an episode of high-grade fever and severe joint pain. The initial symptoms persisted for approximately one week, during which the patient experienced significant discomfort and functional impairment. The patient, an electrical engineer by profession, has no known medical co-morbidities like diabetes mellitus or hypo/hyperthyroidism. Additionally, the patient did not exhibit any skin rash or signs of acromegaly.

Given the patient's history and symptoms, a comprehensive diagnostic workup was initiated. Physical examination revealed classic signs of CTS, including positive Tinel's sign (tingling sensation when tapping over the median nerve at the wrist) and positive Phalen's maneuver (symptoms exacerbated by holding the wrists in flexion). Serological tests confirmed the presence of Immunoglobulin M (IgM) antibodies against CHIKV on two separate occasions, one week apart, indicating a recent CHIKV infection. All other laboratory reports, including comprehensive evaluations of viral markers, thyroid profiles, and additional parameters, were within normal ranges (Tables 1-3).

**Table 1:** Lab reports ESR: Erythrocyte sedimentation rate; CRP: C-reactive protein

Lab Reports	Results	Reference Range
White blood cell count [1000/cmm]	9.4	4-11
Platelet count [1000/cmm]	191	150-450
Red blood cell count [million/uL]	4.89	4.0-5.1
Hemoglobin [g/dL]	11.5	12-14
Mean corpuscular volume [fL]	78.4	80-100
Mean corpuscular hemoglobin [pg]	27.5	27.5-33.2
Mean corpuscular hemoglobin concentration [gm/dL]	33.4	33.4-35.5
Absolute eosinophil count [1000/cmm]	450.91	20-500
Neutrophils [%]	70	40-80
Lymphocytes [%]	20	20-40
Eosinophils [%]	06	1-6
Monocytes [%]	04	2-10
Basophils [%]	00	0-1
ESR [mm/hr]	2	0-20
CRP (mg/dL)	0.2	<0.3

**Table 2:** Biochemistry lab reports

Biochemistry Tests	Results	Reference Range
Blood urea nitrogen (mg/dL)	30	15-40
Creatinine (mg/dL)	1.0	0.5-1.3
Glomerular filtration rate (mL/min/1.73 sq mm)	94	90-120
Total bilirubin (TBI) (mg/dL)	0.8	0.2-1.0
Direct bilirubin (DBI) (mg/dL)	0.2	<0.2
Indirect bilirubin (IBI) (mg/dL)	0.6	0.2-0.8
Aspartate aminotransferase (U/L)	32	15-37
Alanine aminotransferase (U/L)	38	14-59
Alkaline phosphatase (U/L)	91	<98
Lactate dehydrogenase (LDH) (U/L)	200	<250
Total protein (TP) (gm/dL)	7.9	6.4-8.3
Albumin (ALB) (gm/dL)	2.5	3.2-4.5
Sodium (mEq/L)	140	135-145
Potassium (mEq/L)	4.9	3.5-5.0

**Table 3:** Miscellaneous lab reports

Miscellaneous	Results	Reference Range
Anti-nuclear antibody	<1:40	<1:40
Anti-double-stranded DNA (IU/mL)	22	0-25
C3 (mg/dL)	95	90-150
C4 (mg/dL)	20	15-45
Thyroid stimulating hormone (TSH) (mIU/L)	3.5	0.5-5.0
Total T3 levels (ng/dL)	120	80-220
Total T4 levels (mcg/dL)	7.0	5-12

**Figure 1:**
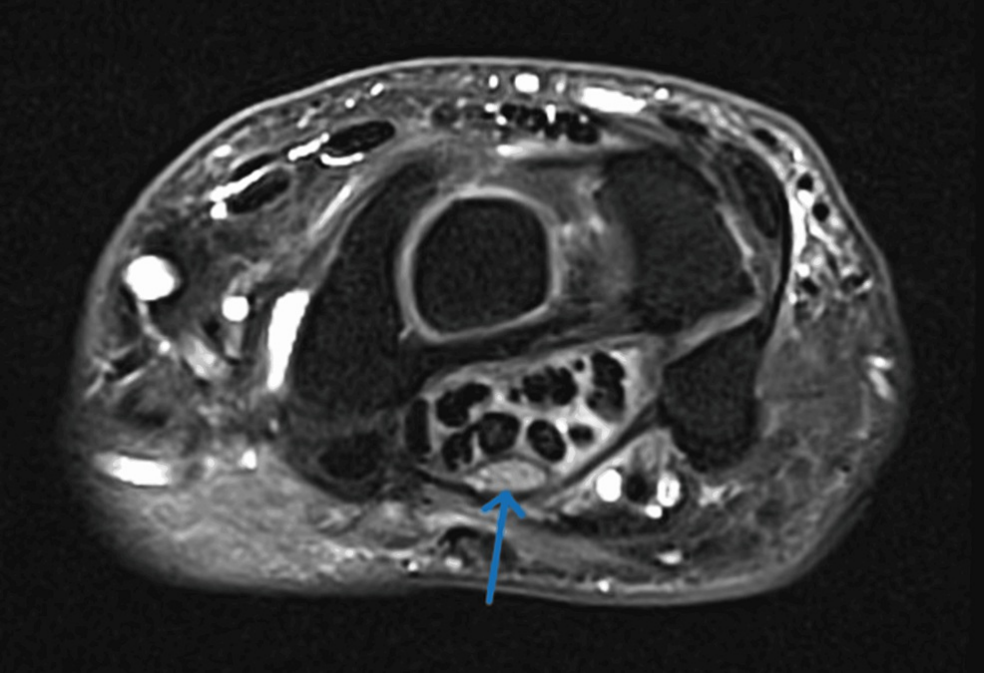
MRI showing median nerve thickening and edema within the carpal tunnel

An MRI of the affected wrist was performed, revealing characteristic features of CTS. The MRI showed palmar bending of the flexor retinaculum, nerve thickening and edema at the intake (pisiform level), increased cross-sectional area, and nerve flattening at the outlet (hook of hamate level). Additionally, edema was noted within the carpal tunnel, confirming the diagnosis of CTS (Figure 1).

The patient was initially treated with an eight-week regimen of corticosteroids aimed at reducing inflammation and alleviating symptoms. Despite this treatment, the patient's motor symptoms showed minimal improvement, indicating that conservative management was insufficient. Due to the lack of significant improvement with corticosteroid therapy, surgical intervention was considered.

The patient underwent carpal tunnel release surgery, which involved cutting the transverse carpal ligament to relieve pressure on the median nerve and expand the carpal tunnel. The surgical intervention led to a successful resolution of symptoms. Postoperatively, the patient experienced significant relief from pain, numbness, and motor impairment. Follow-up assessments indicated improved motor function, with the patient regaining strength and dexterity in the right thumb. The sensory symptoms in the affected hand were markedly reduced, and the patient was able to resume daily activities without significant limitations.

This case highlights the importance of considering CHIKV in the differential diagnosis of CTS, especially in endemic areas. The patient's presentation of CTS following a CHIKV infection underscores the potential for the virus to cause atypical neurological complications. The successful surgical outcome emphasizes the need for an interdisciplinary approach in managing such cases, particularly when conservative treatments fail.

## Discussion

The pathophysiological mechanisms linking CHIKV infection to CTS are complex and multifaceted. One primary mechanism involves the inflammatory response triggered by CHIKV, leading to synovitis and tenosynovitis. These conditions can increase pressure within the carpal tunnel, compressing the median nerve. This inflammatory process is well-documented in CHIKV infections, where cytokines and chemokines are released in response to the virus, resulting in localized swelling and inflammation [1,6]. This case demonstrated classic inflammatory features, evidenced by MRI findings of nerve thickening and edema within the carpal tunnel, correlating well with the synovitis and tenosynovitis expected in such infections.

Another significant aspect is CHIKV's neurotropic properties. The virus can invade neural tissues directly, with viral RNA detected in cerebrospinal fluid and neural tissues of affected individuals [3,7]. This direct invasion can lead to neuropathic damage and contribute to CTS development. The persistence of viral RNA in neural tissues, even after the acute infection phase, suggests that CHIKV can cause prolonged neural inflammation and damage [5]. In this case, the patient’s persistent symptoms despite corticosteroid treatment suggest ongoing neuropathic processes, likely due to direct viral invasion or secondary immune responses targeting neural tissues.

Autoimmune mechanisms may also play a role in CHIKV-associated CTS. The immune response against CHIKV can sometimes become dysregulated, leading to the production of autoantibodies targeting neural tissues. This autoimmune reaction can exacerbate inflammation within the carpal tunnel, further compressing the median nerve and leading to CTS symptoms [7]. The patient’s lack of significant improvement with corticosteroids alone indicates that inflammation may not be the sole mechanism at play; rather, autoimmune processes might contribute to persistent neuropathy.

CTS is primarily characterized by symptoms resulting from the compression of the median nerve as it traverses the carpal tunnel. Typical features include pain, numbness, tingling, and weakness in the hand and fingers, particularly the thumb, index, middle, and radial half of the ring fingers [4]. These symptoms were clearly present in our patient, aligning with the classic presentation of CTS. Atypical features of CTS can arise when it is secondary to conditions such as CHIKV infection. These atypical features include bilateral symptoms, prolonged duration, and resistance to conventional treatments, indicating underlying persistent inflammation or viral effects [8]. Concurrent systemic symptoms such as fever, joint pain, and rash suggest a broader inflammatory or infectious process, unlike isolated idiopathic CTS. In our case, the patient developed CTS symptoms rapidly after CHIKV infection, within weeks, compared to the gradual development seen in idiopathic CTS [9].

Diagnosing CTS in the context of a recent CHIKV infection requires a thorough clinical and diagnostic approach to differentiate it from other causes of median nerve compression. A detailed history should be taken to identify recent CHIKV infection, characterized by fever, rash, and severe joint pain. Physical examination should assess for typical CTS signs, such as positive Tinel's sign and Phalen's maneuver. Serological tests should confirm a recent CHIKV infection, with detection of CHIKV-specific IgM antibodies indicating a recent infection [10]. Electrodiagnostic studies, such as nerve conduction studies and electromyography, are crucial for confirming the diagnosis of CTS in uncertain cases and assessing the extent of nerve damage. Imaging studies, such as ultrasound and MRI, can visualize the structures within the carpal tunnel and detect swelling of the median nerve, tenosynovitis, and other anatomical changes indicative of CTS. Blood tests to measure inflammatory markers, such as C-reactive protein and erythrocyte sedimentation rate, can provide additional information on the inflammatory status of the patient [11].

Several case reports and studies have highlighted the occurrence of CTS following CHIKV infection, providing valuable insights into this rare complication. For instance, a study reported multiple cases of CTS in patients recovering from CHIKV infection, emphasizing the need to consider viral etiologies in patients with atypical CTS symptoms [8]. Similarly, a study described a case where severe tenosynovitis and synovitis following CHIKV infection led to carpal tunnel compression, underscoring the importance of recognizing viral-induced inflammatory conditions as a cause of CTS [9]. A study noted that a small percentage of CHIKV patients developed CTS, highlighting the distinct clinical entity of CTS within the broader spectrum of CHIKV-induced neuropathic pain [10].

The management of CTS associated with CHIKV presents unique challenges due to the need to address both the viral infection and the resultant neuropathic effects. Initial treatment typically comprises NSAIDs, wrist splints, and local steroid injections. In cases of refractory CTS, systemic steroids may also be utilized; however, as seen in the presented case and supported by the literature, corticosteroids alone may not suffice [11]. The persistence of symptoms despite steroid treatment suggests additional factors, such as direct viral damage or autoimmune processes, might be involved.

Surgical intervention, particularly carpal tunnel release surgery, has demonstrated promising results in cases of CHIKV-associated CTS. This procedure aims to relieve pressure on the median nerve by cutting the transverse carpal ligament, thereby expanding the carpal tunnel. The significant symptom relief and functional recovery observed post-surgery in the discussed case align with findings from other studies, highlighting the efficacy of surgical intervention when conservative measures fail [8,9]. The timing of surgical intervention is crucial. A study recommended against early surgery in the presence of active inflammation, suggesting that surgery should only be considered once inflammation has subsided to prevent complications such as algodystrophy [12].

Recognizing the potential for CHIKV to cause CTS is critical for clinicians in endemic areas. Early identification and appropriate management can prevent prolonged disability and improve patient outcomes. Clinicians should maintain a high index of suspicion for viral etiologies in patients presenting with atypical CTS, particularly following a recent febrile illness consistent with CHIKV infection. Diagnostic practices should incorporate serological tests for CHIKV and other relevant viral markers in the evaluation of CTS. Electromyography and nerve conduction studies remain essential for confirming median nerve compression and assessing the severity of nerve involvement.

Future research should focus on elucidating the precise mechanisms through which CHIKV induces CTS. Longitudinal studies tracking patients with CHIKV infection over time could provide valuable insights into the development of CTS and other neurological complications. Additionally, exploring the role of antiviral and immunomodulatory therapies in preventing or mitigating these complications could lead to more effective treatment protocols [7]. Investigating genetic and immunological factors that predispose certain individuals to develop CTS following CHIKV infection could also be beneficial. Identifying potential biomarkers for susceptibility may guide personalized treatment approaches. The development of vaccines and antiviral drugs specific to CHIKV is another critical area of research. While a vaccine (Ixchiq) has been recently approved, ongoing monitoring of its efficacy in preventing neurological complications, including CTS, is necessary [6].

In summary, CHIKV's capacity to cause diverse and atypical complications, such as CTS, highlights the need for comprehensive clinical awareness and a multidisciplinary approach to treatment. By integrating clinical vigilance, timely diagnosis, and collaborative management strategies, healthcare providers can better address the full spectrum of CHIKV-related diseases. Further research will continue to advance our understanding of these complex interactions, ultimately improving care for affected patients.

## Conclusions

This case report underscores the importance of recognizing CHIKV as a potential etiological factor in the development of CTS, particularly in endemic regions. Despite initial conservative treatment with corticosteroids, significant symptom relief and functional recovery were achieved through surgical intervention, highlighting the necessity for considering surgical options when conservative measures fail. This case illustrates the diverse and sometimes unexpected neurological complications of CHIKV, emphasizing the need for an interdisciplinary approach to managing such cases. Enhanced awareness and understanding of the broad spectrum of CHIKV manifestations can lead to more timely and effective treatments, ultimately improving patient outcomes.
